# Brassinosteroids Mitigate Cadmium Effects in Arabidopsis Root System without Any Cooperation with Nitric Oxide

**DOI:** 10.3390/ijms23020825

**Published:** 2022-01-13

**Authors:** Federica Della Rovere, Diego Piacentini, Laura Fattorini, Nicoletta Girardi, Dario Bellanima, Giuseppina Falasca, Maria Maddalena Altamura, Camilla Betti

**Affiliations:** 1 Department of Environmental Biology, Sapienza University of Rome, 00185 Rome, Italy; federica.dellarovere@uniroma1.it (F.D.R.); diego.piacentini@uniroma1.it (D.P.); laura.fattorini@uniroma1.it (L.F.); nicoletta.girardi@outlook.it (N.G.); dario.bellanima@libero.it (D.B.); giuseppina.falasca@uniroma1.it (G.F.); mariamaddalena.altamura@uniroma1.it (M.M.A.); 2 Department of Biosciences, University of Milan, 20133 Milan, Italy

**Keywords:** adventitious roots, auxin localization, brassinazole, CdSO_4_, epibrassinolide, lateral roots, nitric oxide epifluorescence, nitroprusside, quiescent center, root apices

## Abstract

The heavy metal cadmium (Cd) affects root system development and quiescent center (QC)-definition in Arabidopsis root-apices. The brassinosteroids-(BRs)-mediated tolerance to heavy metals has been reported to occur by a modulation of nitric oxide (NO) and root auxin-localization. However, how BRs counteract Cd-action in different root types is unknown. This research aimed to find correlations between BRs and NO in response to Cd in Arabidopsis’s root system, monitoring their effects on QC-definition and auxin localization in root-apices. To this aim, root system developmental changes induced by low levels of 24-epibrassinolide (eBL) or by the BR-biosynthesis inhibitor brassinazole (Brz), combined or not with CdSO_4_, and/or with the NO-donor nitroprusside (SNP), were investigated using morpho-anatomical and NO-epifluorescence analyses, and monitoring auxin-localization by the *DR5::GUS* system. Results show that eBL, alone or combined with Cd, enhances lateral (LR) and adventitious (AR) root formation and counteracts QC-disruption and auxin-delocalization caused by Cd in primary root/LR/AR apices. Exogenous NO enhances LR and AR formation in Cd-presence, without synergism with eBL. The NO-signal is positively affected by eBL, but not in Cd-presence, and BR-biosynthesis inhibition does not change the low NO-signal caused by Cd. Collectively, results show that BRs ameliorate Cd-effects on all root types acting independently from NO.

## 1. Introduction

Brassinosteroids (BRs) are polyhydroxy steroids that control a wide range of plant developmental events, including rhizogenesis and vegetative growth [1]. Since their first discovery in *Brassica napus*, decades of research have pointed out the importance of BRs for many plants morphogenic processes. Furthermore, the severe abnormalities occurring in BR-deficient mutants, together with the positive effects of exogenous BR applications on plant growth, clearly demonstrated the significance of this hormone [2]. BRs are perceived at the plasma membrane by the transmembrane receptor BRASSINOSTEROID INSENSITIVE1 (BRI1) and its paralogs [3]. Upon BR binding to BRI1, the ligand-receptor activation results in a series of transphosphorylation events, which involve numerous co-receptors. Downstream of BRI1 and its co-receptors, the signal is transduced intracellularly until the transcription factors BRI1-EMS SUPPRESSOR1 (BES1/BZR2) and BRASSINAZOLE-RESISTANT1 (BZR1), which ultimately regulate plant development [4].

Exogenous BRs may exert both positive and negative effects on root growth, depending on the applied concentration, with high concentrations resulting in growth inhibitory effects [5]. One of the three most bioactive BRs is 24-epibrassinolide (eBL) [6,7] which was found to promote root elongation in Arabidopsis plants, when applied exogenously at low concentrations [8]. Moreover, in Arabidopsis many sterol- and BR-deficient mutants display reduced lateral root (LR) formation, demonstrating root-specific effects of endogenous/exogenous BRs [9].

The necessity of BRs for plant growth has also been demonstrated by the application of the specific drug brassinazole (Brz), a BR biosynthesis inhibitor which inhibits growth, but whose action is reversed by eBL applications [10]. Although BRs were initially identified based on their growth-promoting activities, they are also now known to be involved in the responses to abiotic and biotic stresses [11]. For example, these steroids induce tolerance to drought and cold stresses in *Arabidopsis thaliana* and *Brassica napus* seedlings [1,7], and stimulate the growth under stress in cereal crops and pepper [12,13]. It has been suggested that the protective properties of BRs against stress occur through a modulation of plant development, even if the convergence between adaptation to stress-response and acquisition of normal growth pattern is still a black box for these compounds. However, also auxins and other signaling molecules, such as nitric oxide (NO), play a role in stress response [4], possibly acting at the convergence between adaptation and stress.

The model dicot plant Arabidopsis has a tap root system with a well-developed primary root (PR) with lateral roots (LRs) and a limited number of adventitious roots (ARs) formed at the hypocotyl base [14,15]. Auxin has been widely demonstrated to positively affect the induction and growth of all these root types in Arabidopsis’s root system [16,17]. Soil pollution caused by heavy metals can have adverse effects on the root system by either restricting or enhancing LRs and/or ARs formation as a stress avoidance/survival response [4]. In accordance, the stress caused by the heavy metal cadmium (Cd) usually represses PR elongation, e.g., in Arabidopsis [18], but can have diverse effects on LRs and ARs, either increasing or decreasing their number depending on the species and the culture/soil conditions [19,20]. The normal formation and activity of the quiescent center (QC) in the root apical meristems is known to be under auxin control [17,21], but Cd disrupts the QC in both PR and LRs/ARs of Arabidopsis [19,22,23], and affects the endogenous IAA distribution in the root apices monitored by the use of a *DR5::GUS* auxin-reporter line [19,24]. Auxin and BRs have overlapping activities with many target genes in common [25]. In Arabidopsis PR, BRs are required to control QC identity in addition to auxin [26,27], and interact with auxin signaling to promote LR growth [9,28]. Moreover, in the same plant, low levels of exogenous BRs promote LR initiation by increasing acropetal auxin transport, whereas at high concentrations suppress LR formation [9]. A role for auxin in protecting the root system from Cd has been demonstrated in rice [29], which was similar to the mitigative effects on Cd accumulation and toxicity observed after exogenous BR applications in other crops [30,31].

Nitric oxide (NO) is a reactive nitrogen species which acts as signaling molecule coordinating development and stress response [32]. In rice ARs and LRs, Cd reduces NO levels, but the NO formed by sodium nitroprusside (SNP) decreases Cd uptake and enhances the NO-levels with this resulting into an alleviation of the morphological alterations induced by the heavy metal [33]. In several plants NO interacts with auxin in PR, LR and AR formation, even under heavy metal stresses [34], and in rice NO counteracts the Cd-effects on auxin distribution [29]. In Arabidopsis, a role of NO and auxin in Cd-mediated root meristem growth has been determined. In particular, the Cd-induced NO accumulation in the root has been shown to repress auxin transport and signaling, with this leading to decreased auxin levels in the apex, with consequent anomalies [35,36].

Brassinosteroids have been reported to exploit NO-mediated mechanisms to provide stress tolerance, e.g., in Arabidopsis, *Cucumis sativus* and maize [37,38,39]. In addition, at least in cucumber, BRs induce endogenous NO formation at the same time promoting AR formation, and the combined application of BRs and an NO-donor compound (S-nitroso-N-acerylpenicillamine) further promotes ARs [40]. However, the link between BRs and NO in root formation under normal and stressed (Cd) conditions still needs investigation [4,40]. It would be important to elucidate part of the mechanisms at the convergence between growth and adaptation to an abiotic stress involving root system plasticity.

Collectively, the aim of our work is to provide evidence that BRs are essential morphogenic/repairing hormonal signals affecting auxin localization and QC definition in the different components of the root system (PR, LRs and ARs) of Arabidopsis in response to Cd, and to determine whether there is a cooperation with the NO-response induced by the same pollutant. Our hypothesis is to establish whether BRs exhibit protective properties against Cd-stress through a modulation of root system development acting as phytohormones with a role in the convergence of adaptation to stress-response.

## 2. Results

### 2.1. Epibrassinolide Enhances Both Lateral and Adventitious Rooting When Applied at Low Concentration, Even in the Presence of Cadmium

**Figure 1:**
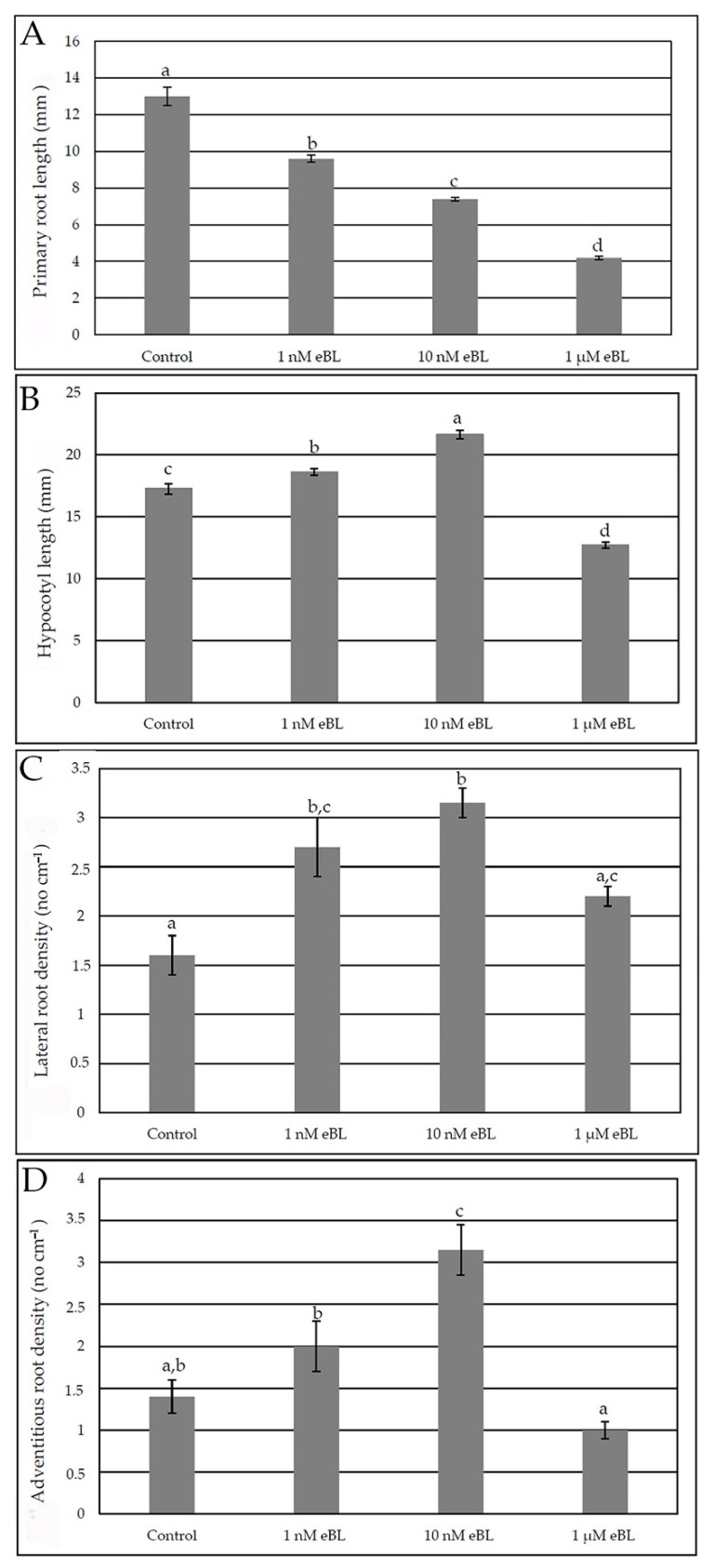
Primary root mean length (±SE) (**A**), hypocotyl mean length (±SE) (**B**), mean density of lateral roots (±SE) (**C**) and mean density of adventitious roots (±SE) (**D**) of Col seedlings cultured for 9 days under continuous darkness followed by 7 days under 16 h light/8 h darkness photoperiod on ½ MS medium (see Section 4) (Control treatment), or on the same medium plus either 1 nM eBL, or 10 nM eBL, or 1 µM eBL. Different letters show significant differences for at least *p* < 0.05. *n* = 30. Data from the first replicate.

Wild-type Arabidopsis seedlings were grown for 9 days in dark regime and then transferred for 7 days under long day photoperiod on media containing different eBL concentrations, i.e., 1 nM, 10 nM, 1 µM. None of the tested eBL concentrations negatively affected seed germination, but seedling growth became stunted in the presence of 1 µM eBL (Figure S1D in comparison with Figure S1A–C, see Supplementary Materials), with hypocotyl length highly reduced (Figure 1B). The root system of the seedlings was carefully analyzed; primary root (PR) elongation was negatively affected by eBL treatments with a significant and progressive reduction with increasing hormone concentrations (Figure 1A). In contrast with the negative effect on hypocotyl elongation caused by the highest eBL concentration, the other two concentrations, and especially 10 nM, significantly increased hypocotyl length in comparison with the Control treatment (Figure 1B).

All the eBL treatments caused significant increases in lateral root (LR) formation (Figure 1C). The concentration of 10 nM, better than that of 1 nM, also significantly increased adventitious root (AR) formation, whereas the concentration of 1 µM was inhibitory and so excluded from the further root morphometric analysis (Figure 1D).

**Figure 2:**
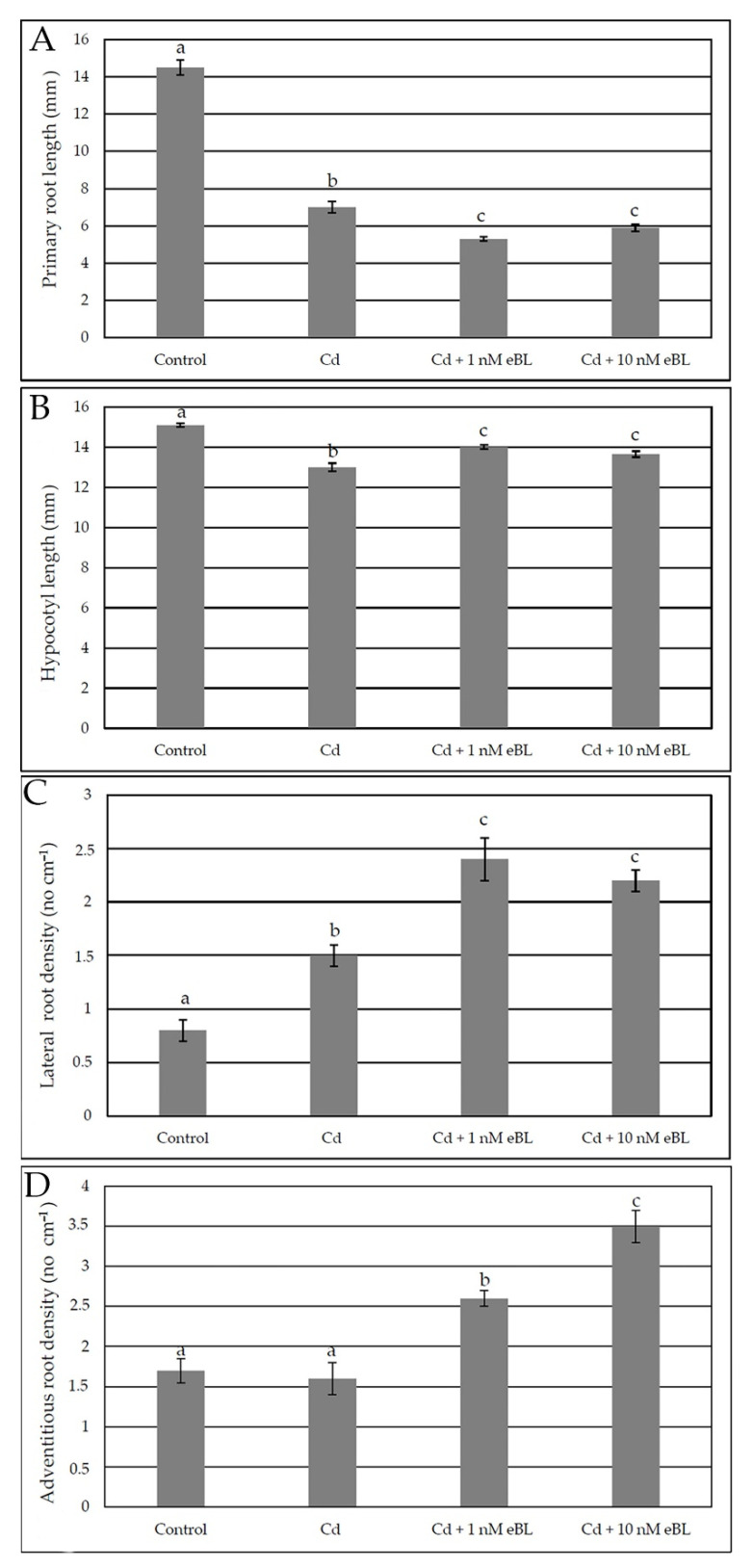
Primary root mean length (±SE) (**A**), hypocotyl mean length (±SE) (**B**), mean density of lateral roots (±SE) (**C**) and mean density of adventitious roots (±SE) (**D**) of Col seedlings cultured for 9 days under continuous darkness followed by 7 days under 16 h light/8 h darkness photoperiod on ½ MS medium (see Section 4) (Control treatment), or on the same medium plus either 60 μM CdSO_4_ (Cd) or Cd + 1 nM eBL, or Cd + 10 nM eBL. Different letters show significant differences for at least *p* < 0.05. *n* = 30. Data from the first replicate.

In accordance with previous data [19,41] the treatment with 60 μM CdSO_4_ reduced to about the half the PR length in comparison with the Control treatment (Figure 2A), and slightly, but significantly, also the hypocotyl length (Figure 2B). The addition of either 1 nM or 10 nM eBL in combination with Cd did not result into a recovery of PR elongation in comparison with Cd alone, and without any difference between the two concentrations (Figure 2A). A slight, but significant, recovery occurred instead in hypocotyl elongation when both the eBL concentrations were combined with Cd (Figure 2B).

A significant increase in LR formation, including both primordia and elongated roots, was caused by Cd in comparison with the Control (Figure 2C), in accordance with previous data [19]. Interestingly, both the eBL concentrations used in combination with the pollutant resulted into a strong increase in LR formation, without significant differences between the two (Figure 2C).

Even if the density of ARs, including both primordia and elongated roots, did not change in the presence of Cd alone in comparison with the Control, both the eBL concentrations, and in particular the 10 nM one, strongly increased it when combined with Cd (Figure 2D).

### 2.2. Epibrassinolide Induces Regular QC Formation in the Root Apices of Cadmium-Cultured Seedlings

**Figure 3:**
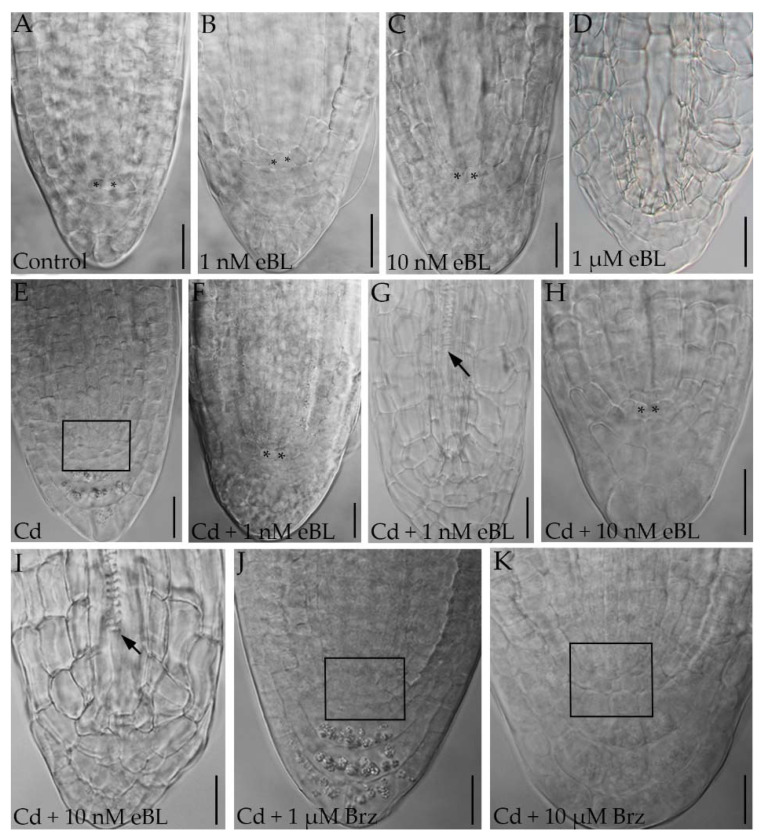
Nomarski images of adventitious root apices of Col seedlings cultured for 9 days under continuous darkness followed by 7 days under 16 h light/8 h darkness photoperiod on ½ MS medium (see Section 4) (Control treatment) (**A**) or on the same medium plus either 1 nM eBL (**B**), or 10 nM eBL (**C**), or 1 µM eBL (**D**), or 60 μM CdSO_4_ (Cd) I, or Cd + 1 nM eBL (**F**,**G**), or Cd + 10 nM eBL (**H**,**I**), or Cd + 1 µM Brz (**J**), or Cd + 10 µM Brz (**K**). The asterisks show the quiescent center (QC) cells (**A**–**C**,**F**,**H**). The rectangles show anomalous cell proliferation instead of QC formation (**E**,**J**,**K**). Arrows show precocious xylem differentiation (**G**,**I**). Bars = 20 µm.

It is known that root growth depends on regular quiescent center (QC) formation and maintenance in the apex [17,42]. The QC is the organizer of the root stem cell niche and its destruction causes differentiation in the stem cells, and anomalous root development [19,43]. As exemplified for ARs (Figure 3), the apical structure of postembryonic roots, which are ARs and LRs in Arabidopsis [17], showed regular QC formation under 1 nM and 10 nM eBL treatments, but irregular cell proliferation, and absence of a QC, under 1 µM eBL (Figure 3D). The latter result was in accordance with the reduction in LR/AR formation observed under the latter treatment (Figure 1C,D). Based on these results, 1 µM eBL concentration was excluded from subsequent experiments except from auxin localization analyses. It is known that the exposure to specific Cd concentrations inhibits PR growth by affecting stem cell niche [22], and that Cd causes impaired QC definition and functioning in LRs and ARs [19]. In accordance with these studies, Cd caused anomalous cell proliferation and no correct QC definition in all the component-types of the root system (Figure 3E, rectangle) but, interestingly, the addition of eBL at 1 and 10 nM together with Cd caused regular QC formation in all root types (Figure 3F,H). Nevertheless, roots showing precocious vascular differentiation and no QC definition were randomly observed (Figure 3G,I). The addition of DMSO did not change Cd response in any root type (Figure S2). When Brz was added with Cd, the PR, LR and AR apical structure did not show a correct QC definition, but rather apical cell proliferation. In ARs grown with the higher Brz concentration, precocious vascular differentiation and cell hypertrophy also occurred (Figure 3J,K). 

Altogether, data with the BR-biosynthesis inhibitor show that the Cd-induced QC aberration does not involve the endogenous production of BRs.

### 2.3. Epibrassinolide Enhances the Auxin Signal in the Root Apices of Cadmium-Cultured Seedlings

**Figure 4:**
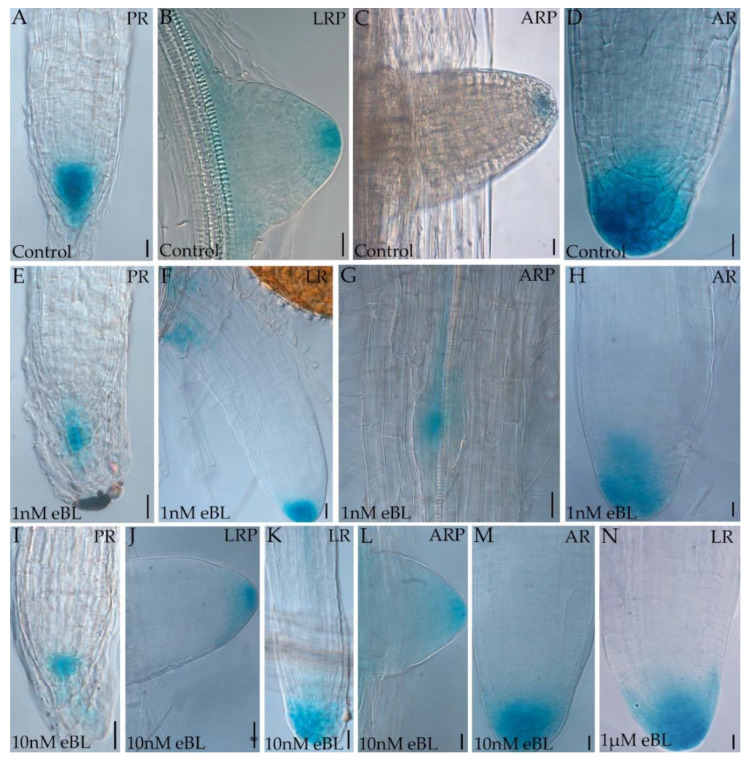
*DR5::GUS* expression in primary roots (PRs) (**A**,**E**,**I**), in adventitious roots (AR) primordia (ARPs, **C**,**G**,**L**), in AR apices (**D**,**H**,**M**), in lateral roots (LR) primordia (LRP, **B**,**J**) and in LRs (**F**,**K**,**N**) of Col seedlings cultured for 9 days under continuous darkness followed by 7 days under 16 h light/8 h darkness photoperiod on ½ MS medium (see Section 4) (Control treatment, **A**–**D**), or on the same medium plus either 1 nM eBL (**E**–**H**), or 10 nM eBL (**I**–**M**), or 1 µM eBL (**N**). Bars: (**A**–**D**,**H**,**L**–**N**) = 10 μm and (**E**,**G**,**I**–**K**) = 20 μm. Images from the first replicate.

**Figure 5:**
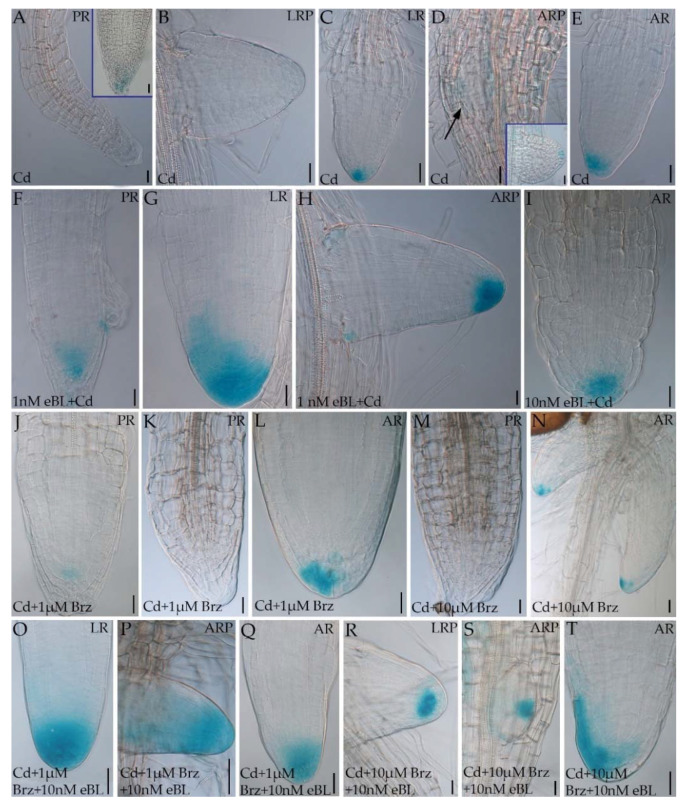
*DR5::GUS* expression in primary roots (PRs) (**A**, and insert, **F**,**J**,**K**,**M**), lateral roots (LR) primordia (LRP, **B**,**R**), LRs (**C**,**G**,**O**), adventitious roots (AR) primordia (ARPs, **D**, arrow and insert, **H**,**P**,**S**), and in AR apices (**E**,**I**,**L**,**N**,**Q**,**T**) of Col seedlings cultured for 9 days under continuous darkness followed by 7 days under 16 h light/8 h darkness photoperiod on ½ MS medium (see Section 4) with either 60 μM CdSO_4_ (Cd, **A**–**E**) or 1 nM eBL + Cd (**F**,**H**), or 10 nM eBL + Cd (**G**,**I**), or 1 µM Brz + Cd (**J**–**L**), or 10 µM Brz + Cd (**M**,**N**), or 1 µM Brz + Cd +10 nM eBL (**O**–**Q**), or 10 µM Brz + Cd +10 nM eBL (**R**–**T**). Bars = 20 μm, inserts in **A** and **D** = 10 μm. Images from the first replicate.

Auxin localization was histologically monitored in the apices of all the root types of the Arabidopsis root system by the use of *DR5::GUS* transgenic seedlings cultured with/without Cd and in the presence/absence of eBL (Figure 4 and Figure 5). In the few roots obtained with 1 µM eBL the signal was strong but diffused all over the apical dome (Figure 4N). By contrast, the other two eBL concentrations caused an auxin localization in the LR and AR apices as in the Controls, whereas the signal in the PR apex was quite weaker than in the Controls (Figure 4A,E,I, in comparison). However, the niche cells surrounding the QC showed the auxin signal in all root types in the presence of eBL (Figure 4). The treatment with Cd alone negatively affected auxin localization in the apices of all root types reducing both the signal and its intensity in the meristem cell area, up to its disappearance in the niche, in surrounding cells, and all around (Figure 5A–E), collectively showing a strong disturbance in auxin apical distribution caused by the pollutant. 

By contrast, the combined presence of the pollutant with either 1 nM eBL or 10 nM eBL highly reinforced the auxin signal (Figure 5F–I) comparably to the effects of eBL alone (Figure 4 and Figure 5 in comparison).

Interestingly, the application of the pollutant combined with Brz (both at 1 µM and 10 µM) did not cause any change in the reduction of the auxin apical signal caused by Cd alone (Figure 5J–N and A–E in comparison), and also in these treatments some PRs showed no auxin signal as under Cd alone (Figure 5K,M and A in comparison). No effect of the Brz solvent DMSO per se was observed when it was combined with Cd (Figure S3A,B). However, when eBL (10 nM) was applied in combination with the BR-biosynthetic inhibitor (at both concentrations) and Cd, the auxin signal increased independently of the root type (Figure 5O–T) becoming similar to that observed under either 1 or 10 nM eBL alone treatments (Figure 4E–M).

Collectively, the results with eBL and Brz treatments show that BRs positively interact with auxin fluxes, and with the definition of the apical auxin maximum, whereas Cd interacts negatively, with this effect mitigated by the exogenous BR application, but not by the endogenous BR synthesis.

### 2.4. Exogenous Nitric Oxide Enhances Lateral and Adventitious Root Formation in the Presence of Cd without Any Synergism with eBL

Because of the higher rooting response caused by 10 nM eBL, in comparison with 1 nM, and considering the similar results in auxin localization (Figure 4), the 10 nM eBL concentration was selected for further experiments. It is known that the NO derived by the exogenous application of the donor compound SNP is able to mitigate the deleterious effects of Cd in rice when applied at 50 μM [33]. By the use of the same SNP concentration, combined or not with either Cd or eBL, the morphology of the root system was analyzed.

**Figure 6:**
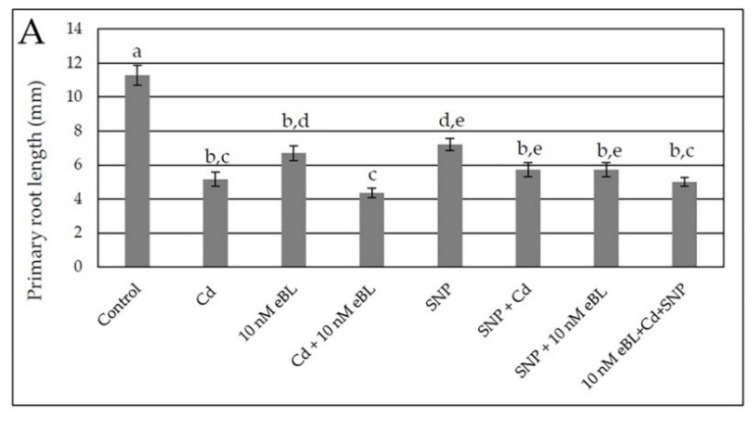

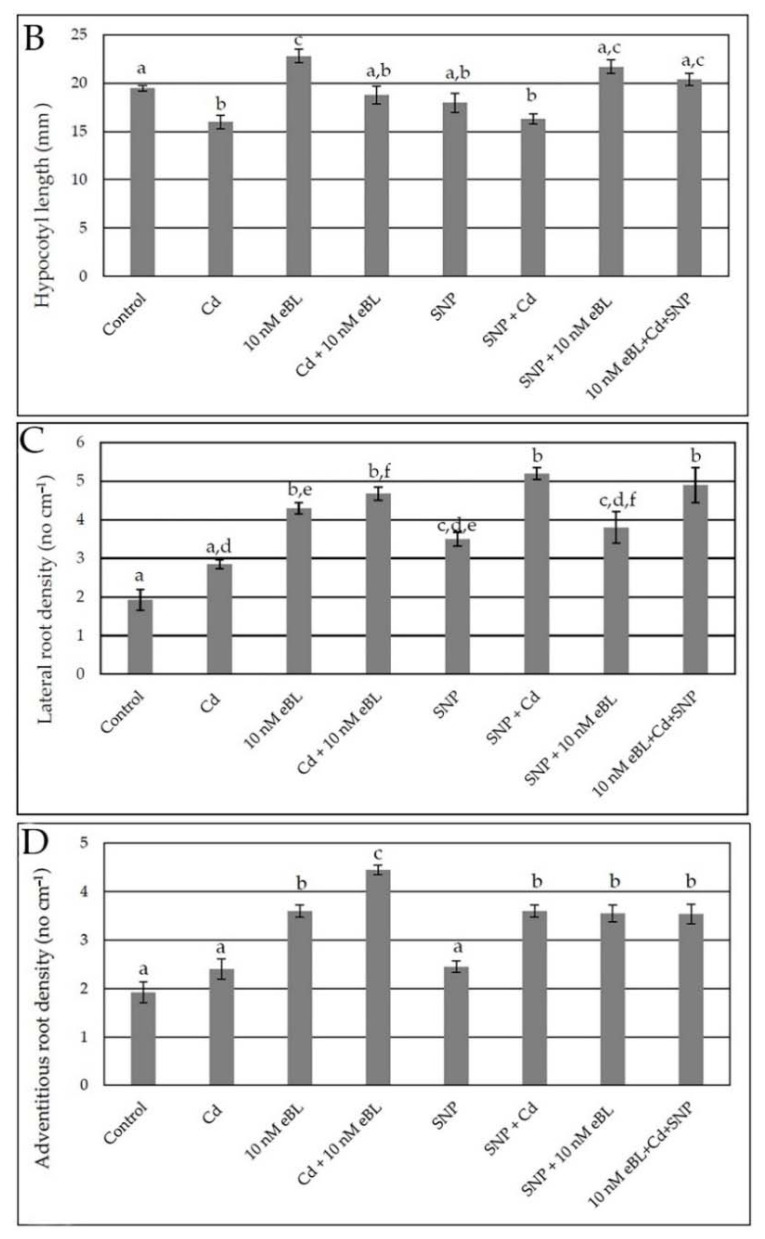
Primary root mean length (±SE) (**A**), hypocotyl mean length (±SE) (**B**), mean density of lateral roots (±SE) (**C**) and mean density of adventitious roots (±SE) (**D**) of Col seedlings cultured for 9 days under continuous darkness followed by 7 days under 16 h light/8 h darkness photoperiod on ½ MS medium (see Section 4) (Control treatment), or on the same medium plus either 60 μM CdSO_4_ (Cd) or 10 nM eBL, or Cd + 10 nM eBL, or 50 μM SNP (SNP), or 50 μM SNP + 60 μM Cd (SNP + Cd), or 50 μM SNP + 10 nM eBL (SNP + 10 nM eBL), or 10 nM eBL + 60 μM CdSO_4_ + 50 μM SNP (10 nM eBL + Cd + SNP). Different letters show significant differences for at least *p* < 0.05. Columns followed by the same letters are not significantly different. *n* = 30. Data from the first replicate.

All treatments significantly (*p* < 0.0001) reduced PR length in comparison to the Controls (Figure 6A), albeit less with eBL or SNP, and no recovery of PR growth was induced by eBL when combined with SNP, with/without Cd, in comparison with the Controls (Figure 6A). Differently, the treatment with the NO donor caused a slight increase in hypocotyl length in comparison with the treatment with Cd alone, reaching the Control values (Figure 6B). The eBL alone treatment continued to cause a significant increase in hypocotyl length in comparison with the Controls (Figure 1B and Figure 6B), but, when combined with the NO donor, with or without Cd, there was no significant change in hypocotyl elongation in comparison with eBL alone (Figure 6B).

The NO donor application resulted into an increase in LR formation which was quite lower than that induced by the eBL alone treatment (Figure 6C). However, no synergic enhancement resulted from their combined application, because LR density remained more similar to SNP alone than to eBL alone (Figure 6C). 

In Cd presence, both eBL and SNP enhanced LR formation, but without a synergistic effect coming from their combined application with the pollutant (Figure 6C). Differently from what observed for LR formation, the treatment with SNP alone did not cause a significant increase in AR formation in comparison with the Control (Figure 6D). However, SNP resulted into a significant increase in AR formation when combined with Cd, as observed for LR formation (Figure 6C,D in comparison). The increase in AR formation due to 10 nM eBL combined with Cd was confirmed (Figure 2D and Figure 6D), but it was not further enhanced in the presence of also SNP, with/without Cd (Figure 6D). The observation that the production of ARs by eBL combined with Cd was the highest demonstrates that eBL is more efficient than SNP in ameliorating AR response in the presence of Cd, whereas their effects are similar in the case of LR formation (Figure 6C,D in comparison). However, there is no synergistic effect coming from the combined application of the BR and the NO donor with the pollutant also in AR formation (Figure 6D).

### 2.5. Epibrassinolide Increases NO Signal but Not in Cd Presence

For a deep insight into the NO role in root formation in the presence of BRs, the epifluorescence signal caused by this free radical was monitored in primordia and root apices of PR, LRs and ARs. The epifluorescence analysis was focused on entire primordia and on apices of mature roots because their meristems are known to be the root regions more affected by either NO [35,44] or BRs (Figure 3), and because they are the site of the auxin maximum localization (Figure 4) governing the root growth.

**Figure 7:**
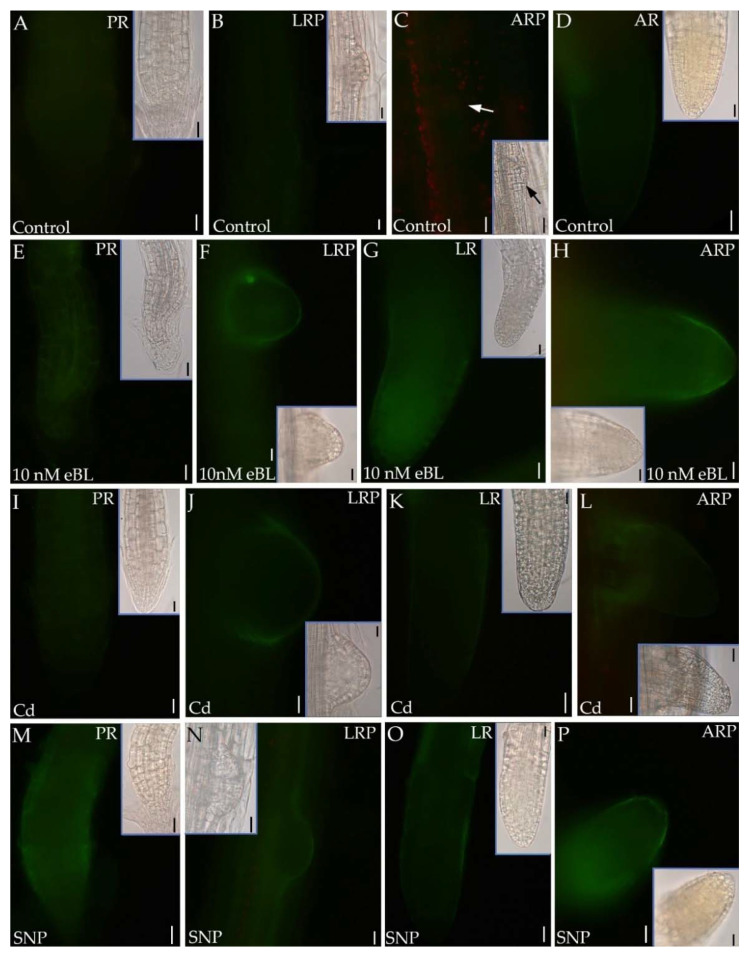
Nitric oxide epifluorescence signal in primary roots (PRs) (**A**,**E**,**I**,**M**), lateral root (LR) primordia (LRP, **B**,**F**,**J**,**N**), adventitious root (AR) primordia (ARP, arrow in **C**,**H**,**L**,**P**), ARs (**D**) and in LRs (**G**,**K**,**O**) of Col seedlings incubated with DAF-FM DA after culture for 9 days under continuous darkness followed by 7 days under 16 h light/8 h darkness photoperiod on ½ MS medium (see Section 4) (Control treatment, **A**–**D**), or on the same medium plus either 10 nM eBL (**E**–**H**) or 60 μM CdSO_4_ (Cd, **I**–**L**) or 50 μM SNP (SNP, **M**–**P**). Inserts are the same apices under white light. Images from the first replicate. Bars: **A**,**B**,**J** and inserts in **A**,**B**,**J** = 10 μm and **C**–**I**,**K**–**P** and inserts in **C**–**I**,**K**–**P** = 20 μm.

**Figure 8:**
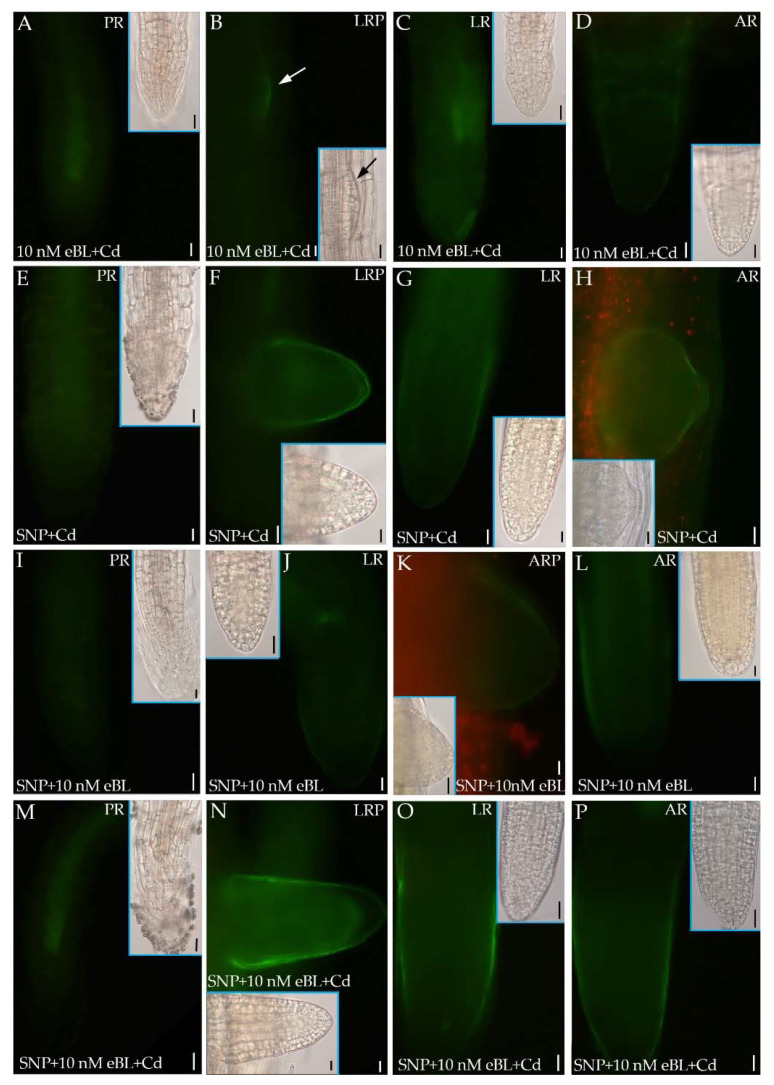
Nitric oxide epifluorescence signal in primary roots (PRs) (**A**,**E**,**I**,**M**), lateral root (LR) primordia (LRP, arrow in **B**,**F**,**N**), LRs (**C**,**G**,**J**,**O**), ARs (**D**,**L**,**P**) and in the adventitious root (AR) primordia (ARP, **H**,**K**), of Col seedlings incubated with DAF-FM DA after culture for 9 days under continuous darkness followed by 7 days under 16 h light/8 h darkness photoperiod on ½ MS medium (see Section 4) plus either 10 nM eBL + 60 μM CdSO_4_ (10 nM eBL + Cd, **A**–**D**), or 50 μM SNP + 60 μM CdSO_4_ (SNP + Cd, **E**–**H**), or 50 μM SNP + 10 nM eBL (SNP + 10 nM eBL**, I**–**L**), 50 μM SNP + 10 nM eBL + 60 μM CdSO_4_ (SNP + 10 nM eBL + Cd, **M**–**P**). Inserts are the same apices under white light. Images from the first replicate. Bars: **A**–**C**,**E**,**J**–**L**,**N**–**P** and inserts in **B**,**G**,**I**,**K** = 10 μm and **D**,**F**–**I**,**M** and inserts in **A**,**C**–**F**,**H**,**J**,**O**,**P** = 20 μm.

**Figure 9:**
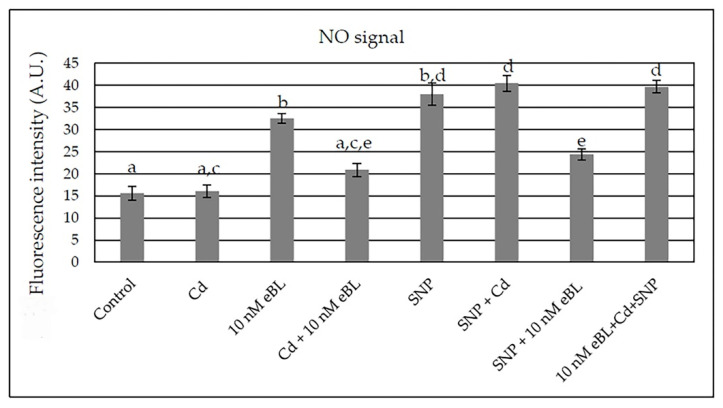
Mean values of nitric oxide (NO) fluorescence intensity (±SE) in Arbitrary Units (A.U.s) in root apices of Col seedlings incubated with DAF-FM DA after culture for 9 days under continuous darkness followed by 7 days under 16 h light/8 h darkness photoperiod on ½ MS medium (see Section 4) (Control treatment), or on the same medium plus either 60 μM CdSO_4_ (Cd) or 10 nM eBL, or Cd + 10 nM eBL, or 50 μM SNP (SNP), or 50 μM SNP + 60 μM Cd (SNP + Cd), or 50μM SNP + 10 nM eBL (SNP + 10 nM eBL), or 10 nM eBL + 60 μM CdSO_4_ + 50μM SNP (10 nM eBL + Cd + SNP). Different letters show significant differences for at least *p* < 0.05. Columns followed by the same letters are not significantly different. *n* = 30. Data from the first replicate.

In comparison with the PR, the LR and AR apical meristems showed a higher NO signal, independently on the treatment (Figure 7 and Figure 8). Moreover, in accordance with the well-known role of NO as compound involved in the perception and response of plants to changing environments, the unstressed Controls showed a very low NO epifluorescence signal (Figure 7A–D). The treatment with eBL (10 nM) alone enhanced the signal in comparison with the Controls (Figure 7A–D,E–H in comparison), with the epifluorescence observations confirmed by the signal quantification (Figure 9). The treatment with Cd alone did not change the NO signal in comparison with the Controls (Figure 7A–D,I–L and Figure 9). The application of SNP alone resulted into an intensified NO signal (Figure 7M–P). However, the quantification of the signal showed the absence of a significant difference with respect to eBL alone (Figure 9). 

The application of eBL with Cd caused a significant reduction in the NO signal in comparison with eBL alone (Figure 8) and this occurred in the apices of the elongated LRs/ARs more than in the primordia (Figure 7E–H and Figure 8A–D in comparison). 

The combination of the NO donor with the pollutant highly increased the signal in comparison with Cd alone (Figure 7I–L and Figure 8E–H in comparison, and Figure 9). By contrast, the combined presence of eBL and SNP reduced the intensity of the NO signal in all root types and developmental stages (Figure 8I–L) in comparison with either eBL or SNP alone (Figure 7E–H,M–P), as also confirmed by the quantification data (Figure 9). The combination of Cd with SNP and eBL reinforced the signal up to values similar to those obtained with SNP combined with Cd (Figure 8E–H,M–P in comparison, and Figure 9), which were significantly higher than those obtained when the BR was combined with Cd in the absence of the NO donor (Figure 9).

### 2.6. The Inhibition of BR Synthesis Does Not Change the NO Signal Caused by Cd

**Figure 10:**
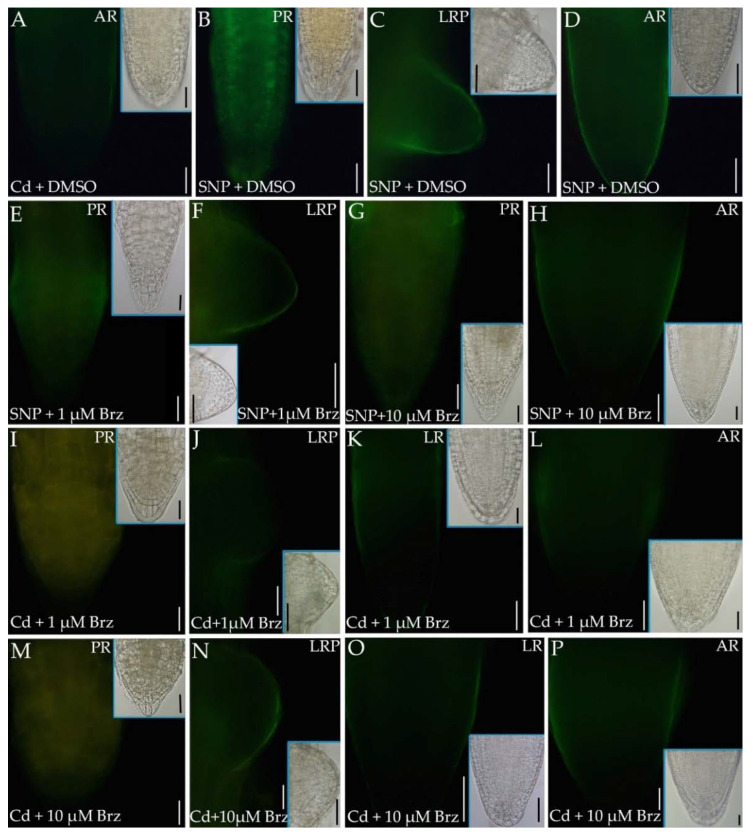
Nitric oxide epifluorescence signal in primary roots (PRs) (**B**,**E**,**G**,**I**,**M**), lateral root (LR) primordia (LRP, **C**,**F**,**J**,**N**), LRs (**K**,**O**), adventitious roots (ARs) (**A**,**D**,**H**,**L**,**P**) of Col seedlings incubated with DAF-FM DA after culture for 9 days under continuous darkness followed by 7 days under 16 h light/8 h darkness photoperiod on ½ MS medium (see Section 4) plus either 60 μM CdSO_4_ + DMSO (Cd + DMSO, **A**), or 50 μM SNP + DMSO (SNP + DMSO, **B**–**D**), or 50 μM SNP + 1 µM Brz (SNP + 1 µM Brz, **E**,**F**), or 50 μM SNP + 10 µM Brz (SNP + 10 µM Brz, **G**,**H**), or 60 μM CdSO_4_ + 1 µM Brz (Cd + 1 µM Brz, **I**,**L**), or 60 μM CdSO_4_ + 10 µM Brz (Cd + 10 µM Brz, **M**,**P**). Inserts are the same apices under white light. Images from the first replicate. Bars = 30 µm.

The epifluorescence signal caused by NO was also monitored in the root apices of PR, LRs and ARs and in LRPs and ARPs after treatments with the NO donor SNP combined with the BR biosynthesis inhibitor Brz at 1 µM and 10 µM. As DMSO is known to negatively affect NO in human cells [45], the possible effect of DMSO on NO production in the root system of Arabidopsis was monitored not only in combination with Cd, but also with SNP. In all root types and stages, the solvent did not change the NO signal detected under Cd (Figure 7I–L and Figure 10A in comparison) or SNP (Figure 10B–D and Figure 7M–P in comparison) treatments, thus demonstrating the absence of an additive/contrasting DMSO effect on NO epifluorescence signal in comparison with Cd or SNP.

The combined application of Cd with each Brz concentration did not affect PR length significantly, but strongly reduced hypocotyl length, with a higher effect induced by the higher Brz concentration (Figure S4A,B). The formation of LRs was unaffected by the Brz and Cd treatment, while ARs formation was affected, with an increased formation in a Brz-concentration-dependent manner (Figure S4C,D). However, independently from these effects on the root system morphology, and considering that both Brz concentrations did not change the Cd-effects on auxin localization (Figure 5A–E,J–N in comparison) and Cd-induced alteration in the QC (Figure 3E,J,K in comparison), both Brz and Cd were applied together with SNP to monitor the effects of BR biosynthesis inhibition on the exogenous NO production. As shown in Figure 10E–H, the inhibition of BR synthesis, without relevant differences between the two Brz concentrations, resulted into an NO-signal comparable to the SNP-induced one, suggesting no synergism between BRs synthesis and NO production in all root types. In accordance, the inhibition of BR synthesis in the presence of Cd did not cause an increase in the NO signal, which remained very low, as with Cd alone or combined with eBL (Figure 7I–L, Figure 8A–D and Figure 10I–P in comparison). However, in a few PRs of the Cd plus Brz treatments, the apex showed a yellowish signal (Figure 10I,M), as a possible consequence of a cell-alteration nullifying the NO specific epifluorescence.

## 3. Discussion

Results show that exogenous BRs stimulate both LR and AR formation, but only when applied at low levels. Exogenous BRs protect the root system from Cd stress by strongly increasing LR and AR formation and growth, and by counteracting its negative effects on QC definition in PR, LRs and ARs. In addition, exogenous BRs, when combined with Cd, revert the negative effects of the pollutant on auxin maximum localization in the apices of all the components of the root system, and the application of the pollutant together with a BR-biosynthesis inhibitor does not result into any reversion of the Cd-induced auxin-localization anomaly. Moreover, BRs positively affect the NO signal, but not in Cd-presence, and no synergism of BRs with the NO derived by the NO-donor SNP occurs in Cd-presence, suggesting that their Cd-protective action is NO-independent.

### Brassinosteroids Protect the Root System against Cadmium-Stress Increasing Lateral and Adventitious Root Formation without Any Cooperation by NO

It is known, and here confirmed, that the stress caused by Cd represses PR-elongation in Arabidopsis [18,19], with the root apex, QC and surrounding cells (in particular) as its preferential target [19,22]. Present data show that Cd causes anomalous cell proliferation, no correct QC definition in the apex of the PR, LRs and ARs, and incorrect auxin maximum localization. However, the application of low levels of BRs nullifies the negative effects of the pollutant on the apical structure and strongly increases LR and AR formation. These events represent an important root system protection by BRs, because the regular apical structure, coupled with an increase in root number, ameliorate the plant efficiency in scouting the soil to search for unpolluted areas.

In Arabidopsis, the positive role of BRs in controlling QC identity in addition to auxin [46] and in interacting with auxin signaling and acropetal auxin transport has been reported [47]. In addition, present data show that, in the absence of stress, specific eBL concentrations do not change auxin localization in the apices of all root types demonstrating that exogenous low levels of BRs do not alter the correct auxin maxima. The situation changes under Cd-stress, because when combined with this pollutant, the same levels of exogenous eBL re-establish the normal acropetal gradient of auxin in the apices altered by Cd. Moreover, the inhibition of BR biosynthesis does not change the alteration in the apical location of auxin caused by Cd. This suggests that under unstressed conditions endogenous BRs must be very low in the root apex, perhaps too low for counteracting root Cd-response. By contrast, under stress conditions different levels of BRs, probably higher than those in unstressed conditions, come into play to restore the normal root system development possibly by interacting with other partners, auxin in particular [48].

Nitric oxide belongs to reactive nitrogen species and, at high concentrations, is known to be a damaging by-product of the plant stress response, including soil pollution response [4]. It has been proposed that Cd induces a significant NO synthesis that contributes to the metal toxicity by favoring inhibition of root growth [18,35]. In Arabidopsis, the accumulation of this free radical induced by Cd represses auxin transport, decreasing auxin levels in the apex, with this resulting into root morphological anomalies [35]. However, NO may also have a positive role on development to counteract stress. Various reports highlight that NO reduces the damages due to abiotic stresses by enhancing the activity of antioxidant enzymes [49,50]. However, its role in the physiological processes depends on its cellular level, functioning either as a signal molecule or as a stress-inducing molecule [51]. In rice ARs and LRs, Cd reduces NO levels, but the NO formed by the NO-specific donor sodium nitroprusside (SNP) decreases Cd uptake and enhances the NO-levels, with this resulting into an alleviation of the morphological alterations induced by Cd [33]. In Arabidopsis, NO contributes to Cd toxicity by promoting Cd accumulation in the roots [18], and jasmonic acid confers plant tolerance to Cd stress by suppressing Cd-induced NO accumulation [52,53]. By contrast, Cd can either increase NO levels, as reported in various plant species [54], or inhibit them [55]. Studies performed in Arabidopsis with the application of NO-specific probes have proven that NO can modulate auxin levels by affecting biosynthesis, degradation, conjugation, distribution, and signaling [56]. Present results show that Cd, at the tested concentration and exposure time, did not significantly change the level of endogenous NO in all root apices in comparison with the unstressed Controls, whereas exogenous NO derived by SNP increases NO levels and improves LR and AR formation in Cd-presence. This supports a developmental role of NO to mitigate Cd effects on the root system.

Brassinosteroids have been reported to utilize NO-mediated mechanisms to provide stress tolerance in Arabidopsis [37]. In cucumber, BRs induce NO formation at the same time promoting AR formation, and the combined application of exogenous BRs and exogenous NO further promotes ARs [40] collectively suggesting a combined role of BRs and NO in promoting rooting. Present data show that low levels of eBL increase LR and AR formation better than SNP, and that there is no further increase in root production by the two in combination. On the other hand, exogenous BRs enhance the endogenous NO production, as shown by the eBL-alone-induced increase in the NO signal, suggesting that the tested BR levels promote root development modulating the endogenous NO levels in the absence of Cd stress. It has been hypothesized that NO is a signaling molecule for plant development at very low levels, and that acts as stress factor at higher concentrations [51]. In accordance, present data suggest that the NO levels required for root development under exogenous BR-control must be very low, because there is no amelioration of the rooting response by the combined application of BRs and exogenous NO, and the fluorescence signal of NO decreases in the eBL + SNP treatment in comparison with eBL or SNP alone. The scenario changes in the presence of Cd stress, because the action of BRs and NO seems to be totally uncoupled. In fact, exogenous BRs stimulate LR and AR formation in the presence of the pollutant similarly to the effects of the NO-donor combined with Cd. Nevertheless, the application of the BRs, the SNP NO-donor and Cd together does not further ameliorate the rooting response, and the epifluorescence NO signal remains the same as after SNP with/without Cd treatments. Moreover, the inhibition of BR biosynthesis in the presence of Cd does not cause any change in the NO signal, which remains very low, as with Cd alone.

In conclusion, by the use of exogenous eBL and treatments with Brz and the NO-donor SNP, our results show that BRs protect the Arabidopsis root system from Cd-stress by counteracting its deleterious morphogenic effects on the apices of all the root types and favoring LR and AR formation. Although BRs are able to positively affect the NO signal, their protective action in response to Cd is independent by NO action.

The knowledge of the mechanisms underlying BR action for regulating AR and LR formation and the relationship with NO will help to understand the link between growth and adaptation in the presence of stress, investigating hormones, e.g., BRs, and signaling molecules, e.g., NO, whose interaction still needs investigation. Bearing in mind that Arabidopsis is a model plant for genetic/physiological/molecular studies, with a lot of genes in common with other plants, the present results offer the possibility of extension to crops of economic value and to providing tools for strategizing new approaches to obtain root systems with efficient abilities to sustain the crop biomass in presence of soil pollutants. 

## 4. Materials and Methods

### 4.1. Plant Material and Growth Conditions

Seeds of *A. thaliana* (L.) Heynh ecotype Columbia (Col) were stratified and sterilized according to [17]. The seeds were sown on a Control medium containing half-strength Murashige and Skoog salts [57], 0.5% sucrose and 0.8% agar, at pH 5.8. The medium was supplemented with 24-epibrassinolide (eBL, Sigma-Aldrich-E1641, Saint Louis, MO, USA) at 1 nM, 10 nM and 1 µM [58]. The stock solution of eBL [10^−4^ M] was obtained by dissolving the hormone in 10% ethanol (EtOH). The EtOH concentration used for obtaining 1 µM eBL, i.e., 0.1% EtOH, was preliminarily tested in the culture medium to verify the absence of negative effects on seed germination and root growth (Figure S5). For Cd treatments, 60 μM CdSO_4_ was also applied alone or combined with 1 or 10 nM eBL. The Cd concentration was chosen according to [19]. Brassinazole (Brz, Sigma-Aldrich-SML1406, Saint Louis, MO, USA), a BR biosynthesis inhibitor, was also used to elucidate the role of endogenous/exogenous BRs on the root system response [58]. The BR inhibitor was applied at 1 µM and 10 µM concentrations [58,59]. DMSO was used as solvent after having tested the absence of negative effects on seed germination and root growth. 

To promote lateral and adventitious root formation, plates containing ten seeds each were placed in vertical position and exposed to white light (100 μEm^−2^s^−1^ intensity) for 6 h, transferred to continuous darkness for 9 days, and then exposed to a 16 h light/8 h dark cycle for further 7 days [19]. Plates were kept in thermostatic greenhouse at 22 ± 2 °C, 70% humidity. Ultrapure water (Milli-Q^®^, Merck, Germany) was used for all culture media. Primary root (PR) length, hypocotyl length, LR and AR density were evaluated in 30 seedlings per experimental replicate and treatment. Hypocotyl and PR length were measured under a LEICA MZ8 stereomicroscope (Leica Microsystems, Germany) using the AxioVision Release 4.7.2 software (Zeiss, Germany) from digital images captured with an AxioCam camera (Zeiss, Germany). Lateral root and AR density were expressed as mean number cm^−1^ (±SE). 

### 4.2. GUS Detection

Seeds of *DR5::GUS* (Col background) transgenic line were sown and grown for 16 days on media supplemented or not with CdSO_4_ and eBL at different concentrations. Thirty randomly selected seedlings per treatment were then processed for β-glucuronidase (GUS) staining according to [60]. Treatments with Brz at 1 µM and 10 µM concentrations, with/without 60 μM CdSO_4_, were also carried out, as well as treatments with 10 nM eBL combined with Brz (1µM and 10 µM) and Cd. DMSO (solvent of Brz) control-treatments in combination with Cd were also carried out (Figure S3). Samples were cleared with chloral hydrate solution [61], mounted on microscope slides and roots were observed with Nomarski optics applied to a Leica DMRB optical microscope (Leica Microsystems, Germany) equipped with a OPTIKA C-P20CC camera (Optika, Italy) to identify quiescent center (QC) organization and auxin maxima in the apices.

### 4.3. Rooting in Response to Exogenous NO and NO Detection in Root Apices

Several treatments were used: 10 nM eBL, alone or combined with Cd; Cd, alone or combined with 50 µM SNP (#71778, Sigma-Aldrich, Saint Louis, MO, USA) [62]; eBL, combined with SNP with/without Cd. The treatment with SNP at 50 µM was also carried out because of its ability to increase intracellular NO levels in rice without causing deleterious effects per se [33]. Treatments with Brz at 1 µM and 10 µM +/−Cd/SNP were also used to elucidate the possible involvement of endogenous BRs in relation with NO endogenous/exogenous content. The morphogenic effects of both Brz concentrations on the root system in the presence of Cd were also verified (Figure S4).

Primary root length, hypocotyl length, and LR and AR density were evaluated in 30 seedlings per experimental replicate and treatment. The NO content was determined in PR, LR and AR apices and primordia by using the specific NO fluorescent probe 4-amino-5-methylamino-2′,7′-difluorofluorescein diacetate (DAF-FM DA, Sigma-Aldrich, Saint Louis, MO, USA) according to [63]. After incubation, the roots were washed three times with fresh 20 mM HEPES/NaOH buffer at pH 7.4 to remove the excess of the probe and observed under a Leica DMRB optical microscope (Leica Microsystems, Wetzlar, Germany) using filters with excitation at 490 nm and emission at 515 nm, equipped with OPTIKA C-P20CC camera (Optika, Italy) and the relative fluorescence quantified using ImageJ software, V1.52a [64] as described for the same plant [65].

### 4.4. Statistical Analysis

Statistical analysis was performed using one way ANOVA test followed by Tukey’s post-test through GraphPad Prism 6.07 software. A normality test (Kolmogorov–Smirnov) was applied before analysis of variance (GraphPad Instat 3). Three independent biological replicates with 30 seedlings each per treatment were carried out with similar results. Data of the first or third replicate were shown.

## Data Availability

Not applicable.

## Supplementary Materials

The following are available online at https://www.mdpi.com/article/10.3390/ijms23020825/s1.
