# Supplementary material for: Brassinosteroids Mitigate Cadmium Effects in Arabidopsis Root System without Any Cooperation with Nitric Oxide
Source: Int J Mol Sci. 2022 Jan 13;23(2):825. doi: 10.3390/ijms23020825 (PMC8776143; doi:10.3390/ijms23020825)
Supplement: Supplementary file 1 [file ijms-23-00825-s001.zip › ijms-1545990-supplementary.pdf]

International Journal of
Molecular Sciences

MDPI

Brassinosteroids Mitigate Cadmium Effects in Arabidopsis Root System without Any Cooperation with Nitric Oxide

Federica Della Rovere [1], Diego Piacentini [1], Laura Fattorini [1], Nicoletta Girardi [1], Dario Bellanima [1], Giuseppina Falasca [1], Maria Maddalena Altamura [1] and Camilla Betti [2,*]

[1] Department of Environmental Biology, Sapienza University of Rome, 00185 Rome, Italy; federica.dellarovere@uniroma1.it (F.D.R.); diego.piacentini@uniroma1.it (D.P.); laura.fattorini@uniroma1.it (L.F.); nicoletta.girardi@outlook.it (N.G.); dario.bellanima@libero.it (D.B.); giuseppina.falasca@uniroma1.it (G.F.); mariamaddalena.altamura@uniroma1.it (M.M.A.)

[2] Department of Biosciences, University of Milan, 20133 Milan, Italy

* Correspondence: camilla.betti@unimi.it

Supplementary materials

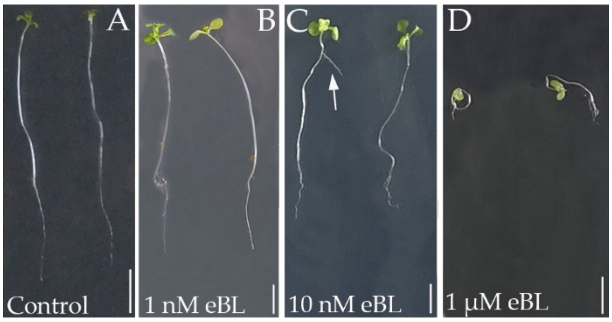

Figure S1. Stereomicroscope images of *A. thaliana* seedlings (Col ecotype) cultured for 9 days under continuous darkness followed by 7 days under 16 h light/8 h darkness photoperiod on ½ MS medium (see Section 4) (Control treatment, **A**), or on the same medium plus either 1 nM eBL (**B**), or 10 nM eBL (**C**), or 1 μM eBL (**D**). Arrow shows the presence of an already elongated adventious root along the hypocotyl. Bars = 5 mm.

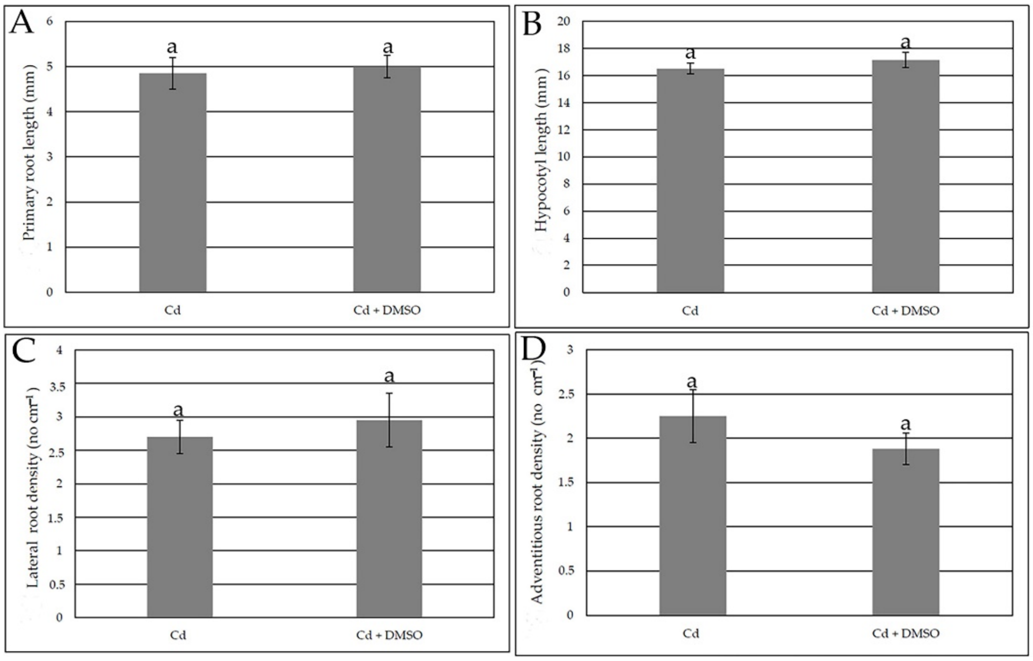

Figure S2. Primary root mean length (±SE) (**A**), hypocotyl mean length (±SE) (**B**), mean density of lateral roots (±SE) (**C**) and mean density of adventitious roots (±SE) (**D**) of *Arabidopsis thaliana* Col–0 seedlings cultured for 9 days under continuous darkness followed by 7 days under 16 h light/8 h

darkness photoperiod on 60 μM $CdSO_4$ (Cd) or on 60 μM $CdSO_4$ (Cd) plus DMSO in the same quantity added for obtaining the 10 μM Brz medium. Columns followed by the same letters are not significantly different. N = 30. Data from the third replicate.

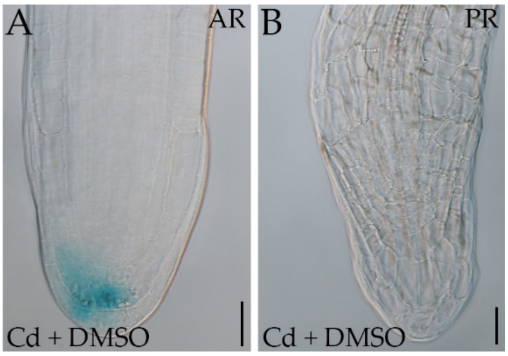

Figure S3. *DR5::GUS* expression in adventious roots (AR) (**A**) and in primary roots (PR) apices (**B**) of Col seedlings cultured for 9 days under continuous darkness followed by 7 days under 16 h light/8 h darkness photoperiod on ½ MS medium (see Section 4) and treated with 60 μM $CdSO_4$ (Cd) combined with the DMSO, solvent of Brz. Bars = 20 μm.

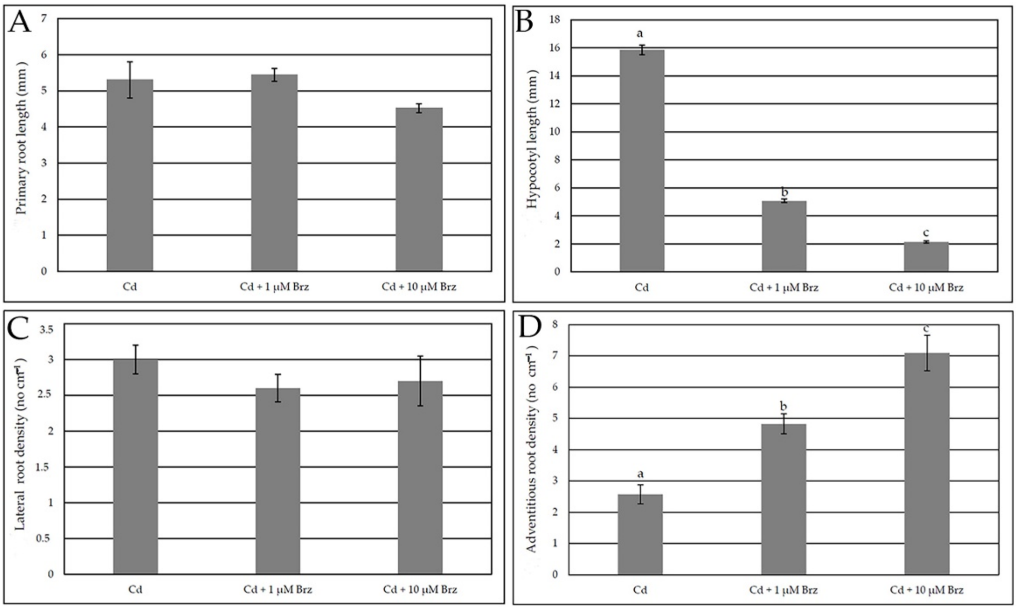

Figure S4. Primary root mean length (±SE) (**A**), hypocotyl mean length (±SE) (**B**), mean density of lateral roots (±SE) (**C**) and mean density of adventitious roots (±SE) (**D**) of *Arabidopsis thaliana* Col seedlings cultured for 9 days under continuous darkness followed by 7 days under 16h light/8 h darkness photoperiod on 60 μM $CdSO_4$ (Cd) or on 60 μM $CdSO_4$ (Cd) + 1 μM Brz, or 60 μM $CdSO_4$ (Cd) + 10 μM Brz. Different letters show significant differences for P<0.0001. Columns followed by no letter are not significantly different. N = 30. Data from the third replicate.

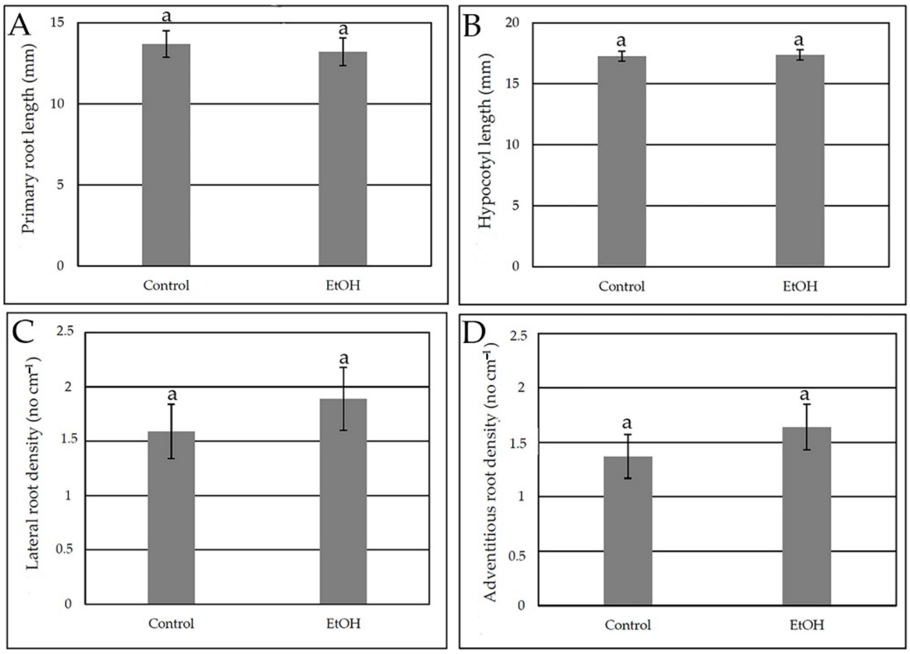

Figure S5. Primary root mean length (±SE) (**A**), hypocotyl mean length (±SE) (**B**), mean density of lateral roots (±SE) (**C**) and mean density of adventitious roots (±SE) (**D**) of *Arabidopsis thaliana* Col seedlings cultured for 9 days under continuous darkness followed by 7 days under 16 h light/8 h darkness photoperiod on ½ MS medium (see Materials and Methods) (Control treatment) or on the same medium plus 0.1% EtOH, i.e. the highest EtOH concentration in the culture media containing eBL. Columns followed by the same letters are not significantly different. N=30. Data from the first replicate.
